# A Pediatric Case of Rapidly Progressing Disseminated Human Adenovirus C1 Infection with Multiorgan Failure and Evidence of Intra-Host Variation

**DOI:** 10.3390/v18060607

**Published:** 2026-05-26

**Authors:** William Otto, Lindsey Rickerman, Maria Deza Leon, Felicia Scaggs Huang, Krithivasan Sankaranarayanan, Christopher Dandoy, Daryl M. Lamson, Adriana E. Kajon

**Affiliations:** 1 Cincinnati Children’s Hospital Medical Center, Cincinnati, OH 45229, USA; william.otto@cchmc.org (W.O.); mpdezaleon@cmh.edu (M.D.L.); felicia.scaggshuang@cchmc.org (F.S.H.); christopher.dandoy@cchmc.org (C.D.); 2 Department of Pediatrics, University of Cincinnati College of Medicine, Cincinnati, OH 45229, USA; 3 Wadsworth Center, New York State Department of Health, Albany, NY 12208, USA; lindsey.rickerman@health.ny.gov (L.R.); krithivasan.sankaranarayanan@health.ny.gov (K.S.); daryl.lamson@health.ny.gov (D.M.L.); 4 Lovelace Biomedical Research Institute, Albuquerque, NM 87108, USA

**Keywords:** adenovirus, hemophagocytic lymphohistiocytosis, next generation sequencing, disseminated disease, intra-host genetic variation, iSNV

## Abstract

A strain of human adenovirus type C1 was isolated from multiple anatomical compartments in a pediatric patient with late-onset Pompe disease. Over a period of 23 days from the appearance of fever and respiratory symptoms until death, disease progression was rapid with severe disseminated disease and complications that included respiratory distress, liver failure, cardiac dysfunction, and hemophagocytic lymphohistiocytosis. Detected viral DNAemia peaked at log_10_ 9.52 copies/mL on the last hospitalization day. Next-generation whole-genome sequencing with depth > 2700 reads/position identified the virus as closely related to the prototype strain Adenoid 71 isolated in the US in 1953, and to strains circulating worldwide in recent years. Sequence data analysis revealed the presence of intra-host single nucleotide variants (iSNV) at low frequency in the isolates recovered from a nasopharyngeal swab, blood, urine, and stool specimens obtained during the last three days of life and from lung, liver, and kidney tissue obtained at autopsy. Evidence of iSNVs was found in only three coding regions (E1A, DNA polymerase, and pVII). Different variant combinations were found in different anatomical compartments. The contribution of intrahost genetic diversity to HAdV-associated disease development and progression warrants investigation.

## 1. Introduction

Human adenoviruses (HAdVs) are double-stranded DNA viruses, well-recognized as prevalent causative agents of acute respiratory illness in both children and adults. Among the 117 recognized genotypes classified within seven species A through G (http://hadvwg.gmu.edu), types 1, 2, and 5 of species C (*Mastadenovirus caesari*) and types 3 and 7 of species B (*Mastadenovirus blackbeardi*) are reported as the most frequently detected in association with pediatric acute respiratory illness in children under five years of age worldwide [1,2,3,4,5,6,7]. While infection frequently has serious consequences in immunocompromised individuals, such as transplant recipients [8,9], disease is usually mild and self-limiting in immunocompetent patients. However, respiratory infection can occasionally result in pneumonia of variable severity or become disseminated with extrapulmonary manifestations [10,11]. Other complications of adenovirus respiratory infection include hemophagocytic lymphohistiocytosis (HLH) and multiorgan system failure [12,13,14,15,16,17,18,19,20,21], but data on type (or species)-specific pathogenicity in the setting of HLH and multiorgan failure remain limited.

In this paper, we describe the clinical and virologic findings in a case of fatal disseminated HAdV infection presenting with HLH-like illness in a pediatric patient with late-onset Pompe disease.

## 2. Materials and Methods

### 2.1. Virus Isolation and Initial Molecular Typing

Whole blood specimens collected on admission to Cincinnati Children’s Hospital Medical Center (CCHMC) and on the following three days of hospitalization, a nasopharyngeal swab (NPS), urine specimen, and stool specimen were also collected on day one of hospitalization, and liver, kidney, and lung tissue obtained from autopsy tested positive by qualitative PCR and were sent to Lovelace Biomedical Research Institute (LBRI). At LBRI, all clinical specimens were processed for virus isolation and initial molecular typing as previously described [22,23]. Isolates were further amplified for one additional passage for the extraction of highly pure intracellular viral DNA as previously described [24]. DNA preparations were examined by horizontal gel electrophoresis after digestion with BamHI (New England Biolabs, Ipswich, MA, USA) for a preliminary species identification. Initial molecular typing was carried out by PCR amplification and Sanger sequencing of the hexon hypervariable regions 1–7 and the fiber gene as previously described [25,26]. Aliquots of all clinical specimens were sent to Wadsworth Center, New York State Department of Health (NYSDOH) in NUCLISENS Lysis buffer (bioMerieux, Marcy-l’Etoile, France) for DNA extraction and assessment of viral load by quantitative real-time PCR (qPCR) as previously described [23]. Highly pure viral DNA aliquots were also sent to NYSDOH for next-generation whole-genome sequencing (WGS).

### 2.2. Next-Generation Whole-Genome Sequencing, Assembly, and Annotation

Purified viral DNA preps from all isolates at passage 2 in A549 cell culture were processed for next-generation sequencing (NGS) in an Illumina MiSeq instrument (Illumina, San Diego, CA, USA) as previously described [27]. The raw fastq reads for each sample were processed using AdapterRemoval v2.3.3 to filter low-quality reads (q < 30), low-quality bases, and remove adapter sequences [28]. The cleaned reads from all eight sequence libraries were mapped against the HAdV-C1 reference genome (AC_000017.1), as well as combined into one set of paired-end reads and used as input for *de novo* assembly. *De novo* genome assembly was performed using the Shovill pipeline v1.1.0 [29], which included read error correction (Lighter v1.1.1) [30], assembly (SPAdes v3.15.5) [31], and assembly improvement (Pilon v1.24) [32]. The resulting genomic sequence was annotated using VAPiD [33].

The cleaned, paired-end reads were mapped against the *de novo* reconstructed adenovirus genome using BWA-MEM v0.7.17 [34]. SAMtools v1.17 [35] and GATK v.4.4 [36] were used to sort the resulting alignments and remove duplicate reads.

### 2.3. Sequence Data Analysis

For phylogenetic analysis, the novel whole-genome sequences were compared to the sequences available in GenBank for various clinical isolates of HAdV-C1 described in Table 1.

Whole genome sequences were aligned in Geneious Prime 2025.0.2. (https://www.geneious.com) using MAFFT and a maximum-likelihood phylogenetic tree was inferred using the Kimura 2-parameter model in iQTree v2.0 [37,38] with the following parameters: GTR + F + I + G4 -b 100. The final tree was visualized in MEGA 11 [39].

Intra-host single nucleotide variant (iSNV) analysis was performed using three approaches: Mutect2 (implemented in GATK v4.4; https://gatk.broadinstitute.org/hc/en-us/articles/360036485152-Mutect2, accessed on 25 September 2025), LoFreq v2.1.5 [40], and Geneious Prime. The following criteria were utilized for stringent iSNV calling/identification: base quality score > 30, mapping quality ≥ 60, strand bias filters, and a minimum minor allele frequency threshold of 3%. Effects of iSNVs were annotated using SnpEff with default parameters [41].

Sequence data for the eight recovered clinical isolates were deposited under BioProject accession number PRJNA1046048 in the NCBI BioProject database (https://www.ncbi.nlm.nih.gov/bioproject, accessed on 1 May 2026).

## 3. Results

### 3.1. Case Description

A four-year-old male with a history of late-onset glycogen storage disease type II presented to his local hospital with a seven-day history of fever, congestion, and cough, as well as abdominal pain, vomiting, and diarrhea. He was admitted to the hospital for the management of dehydration. He continued to have significant diarrhea and was transferred to the pediatric intensive care unit (PICU) for uncompensated shock. He was given broad-spectrum antimicrobials, including vancomycin, cefepime, and metronidazole. Molecular testing of a respiratory specimen using a respiratory pathogen PCR panel revealed infection with both adenovirus and rhinovirus/enterovirus, while testing of stool identified adenovirus and *Giardia duodenalis*. He had no discernible symptoms of Pompe disease and was not on treatment at the time of his adenoviral infection. During hospitalization, he continued to have fevers and developed progressive pancytopenia. He also had liver dysfunction with hepatitis and coagulopathy. Due to these findings, as well as increasing inflammatory markers, there was concern for HLH triggered by the detected infections. He underwent bone marrow aspiration and biopsy, which revealed significant hemophagocytosis, but was negative for viral inclusions. Flow cytometry did not identify malignancy. He was then given pulse methylprednisolone (30 mg/kg) daily for three days. Anakinra, an interleukin-1 receptor antagonist (IL-1Ra), was also administered, initially at 4 mg/kg daily and subsequently at 8 mg/kg. He also received one dose of 400 mg/kg of intravenous immunoglobulin (IVIG). After seven days of hospitalization, his systemic steroids were transitioned to dexamethasone (10 mg). The patient continued to have fevers; hence, plans were made to initiate etoposide. However, he had respiratory distress, worsening hyperferritinemia, and evidence of acute liver failure. Given the severity of his illness, the patient was transferred after 14 days to a quaternary pediatric referral center for further evaluation and management. At admission, the child had a white blood cell count of 3.83 × 10^3^ cells/μL, with hemoglobin 9.2 gm/dL and a platelet count of 38,000 cells/μL. He had normal renal function but significant transaminitis (aspartate transaminase 2893 IU/L, alanine transaminase 391 IU/L), as well as significantly elevated direct bilirubin (7.2 mg/dL) and total bilirubin (11.1 mg/dL). His ferritin level was >99,000 ng/mL. There was concern that his clinical illness was due to disseminated adenovirus infection. He was given cidofovir, dosed at 1 mg/kg three times weekly to avoid renal injury. qPCR testing of blood for HAdV revealed a viral load that was higher than the reportable range of the assay of 2 × 10^9^ genome copies (gc)/mL. Repeated testing of blood specimens on the following two days again identified viral loads above the range. Testing of stool, respiratory, and urine specimens also yielded positive results for HAdV.

The patient progressively worsened and was intubated and mechanically ventilated. He had cardiac dysfunction and required vasopressor support. He received an additional dose of cidofovir (5 mg/kg). He also received an infusion of allogenic HLA class II-matched adenovirus virus-specific T cells (VSTs) produced by stimulation with oligopeptides derived from the hexon capsid protein of HAdV type B3 and the penton base capsid protein of HAdV type C5 [42]. Despite these treatments, the patient continued to worsen and developed abdominal compartment syndrome. The family elected not to continue to escalate care, and the patient died two days after admission to the referral hospital (~ day 24 of illness). Autopsy studies identified the presence of adenovirus in renal, liver, and lung tissue.

### 3.2. Virology Investigation and Findings

qPCR assessment of blood, urine, stool, and respiratory specimens, as well as liver, lung, and kidney tissue homogenate supernatants, confirmed the original testing results and diagnosis of disseminated infection, revealing extremely high viral loads in all examined samples (Table 2). Infectious virus isolates were readily recovered from all received specimens and genetically characterized at passage 2. Restriction enzyme analysis of genomic DNA extracted from infected A549 cell monolayers identified the isolated strain as species *Mastadenovirus caesari* (HAdV-C). PCR amplification and Sanger sequencing of the hexon hypervariable regions 1–7 and of the fiber gene confirmed the molecular identity of the isolates as having a type C1 hexon (H1) and type C1 fiber (F1).

### 3.3. Whole Genome Sequence Data Analysis

The mean sequencing depths for the eight genomes ranged from 2754 reads/position for the respiratory isolate to 3984 reads/position for the urine isolate (Table 3). Following quality filtering and adapter trimming, more than 99% of read pairs were retained in all eight libraries (99.5% ± 0.1%, mean ± s.d.). These analysis-ready reads were used for *de novo* and reference-based assembly and variant determination. The adenovirus genome was assembled into a single contig of 35,988 base pairs. All sequence data were deposited at the NCBI/BioProject database under accession number PRJNA1046048. A BLASTN v2.17.0 (Basic Local Alignment Search Tool-Nucleotide; https://blast.ncbi.nlm.nih.gov/) search for the assembled genomic sequence identified the prototype strain of HAdV-C1 (AC_000017.1) as the closest match with 99.44% identity.

**Figure 1:**
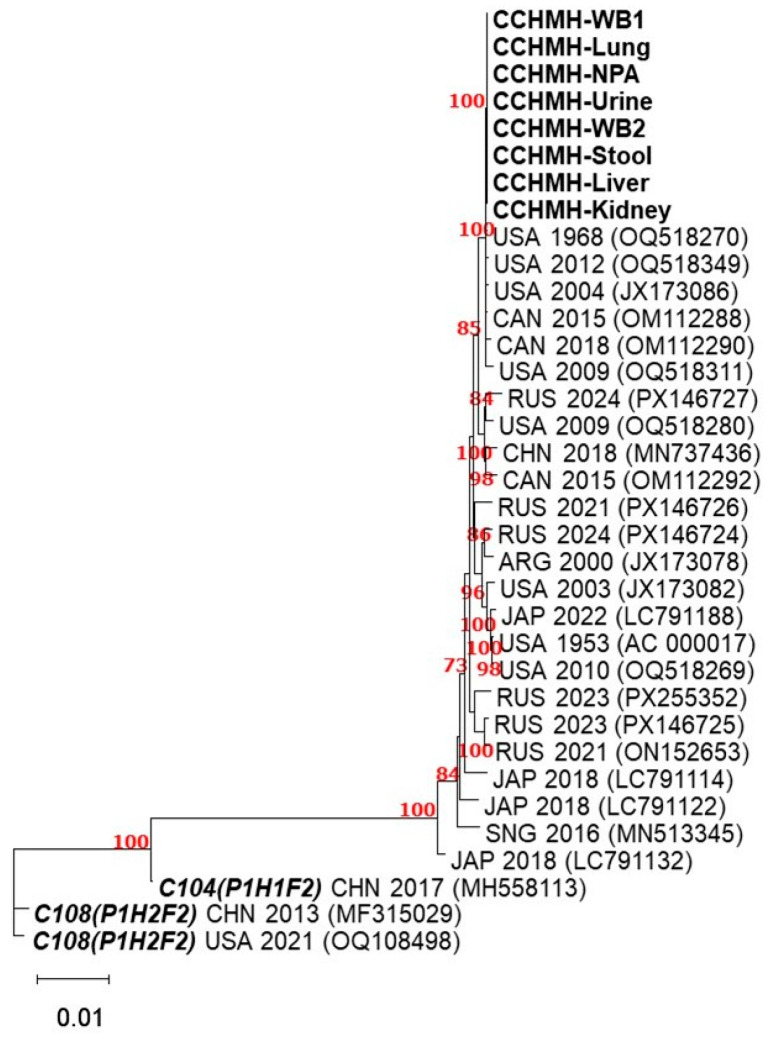
Maximum-likelihood phylogenetic tree of HAdV-C1 consensus whole genome sequences of the eight clinical isolates obtained in this study and 24 selected closely related C1 strains. C104 and C108 genomes were included as a reference in the analysis. The tree was constructed and visualized in MEGA 11. Bootstrap values > 70 are shown in red font. Bootstrap values < 70 are not displayed on the tree.

Based on the results of the genetic characterization, the strain isolated from the patient was designated Human adenovirus C/USA/CCHMC 10-22/2022/P1H1F1. As shown in Figure 1, the phylogenetic analysis of evolutionary relationships and sequence similarities to other HAdV-C1 strains identified the clinical isolates as being most closely related to strains isolated in the USA in 1968, 2012, and 2004, with percent sequence identities of 99.93%, 99.92%, and 99.91%, and 28, 32, and 44 nucleotide differences, respectively. Among the examined strains, the most distantly related was Kobe180476 (LC791132) isolated in Hyögo prefecture, Japan, in 2018 with 99.22% sequence identity (294 nucleotide differences).

**Figure 2:**
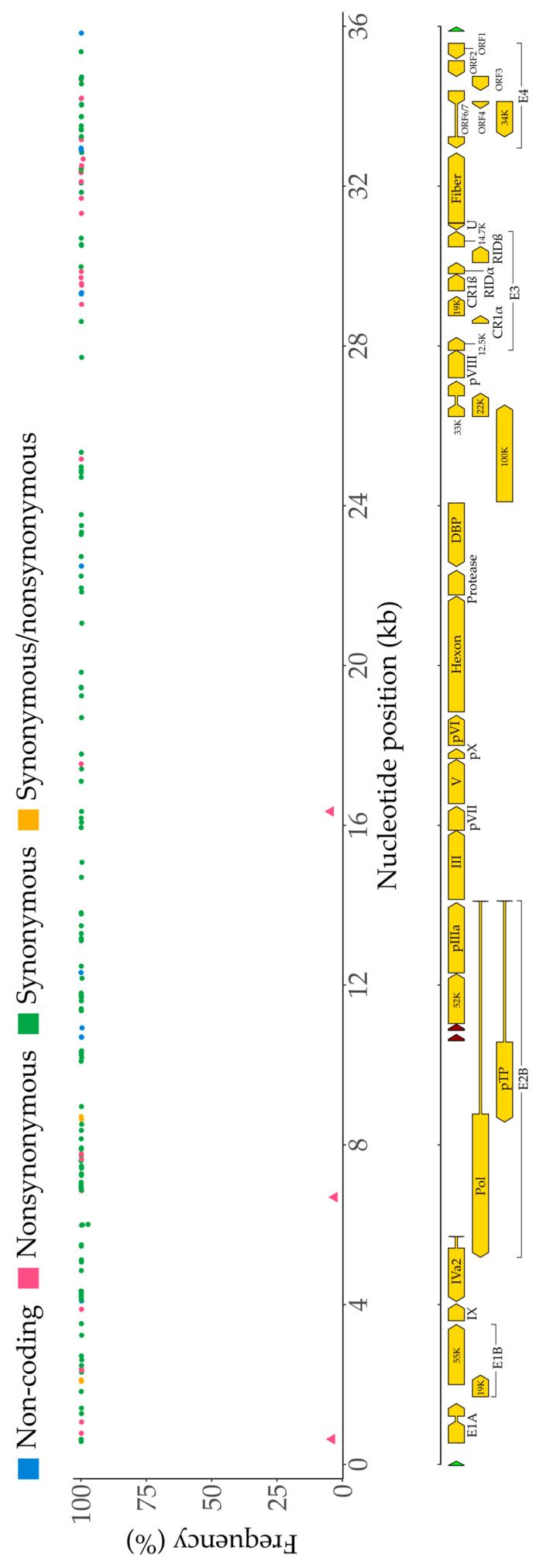
Mean variant allele frequency within the adenovirus genome from passage 2 patient samples. In total, 170 nucleotide positions were present within eight different cultured sample types from one patient, as aligned to the human adenovirus type 1 NCBI reference AC_000017 genome. Triangles denote iSNVs identified in the clinical isolates as described in Table 5. Nucleotide positions 2080, 2115, 8639, and 8705 are labeled ‘Synonymous/nonsynonymous’ because they intersect multiple CDS.

A total of 168 variant sites were consistently identified above the frequency threshold of 3% by the three variant calling approaches when the isolate genomic sequences were compared to the reference HAdV-C1 strain Adenoid 71 isolated in the USA in 1953 (GenBank # AC_000017.1) (Figure 2). Out of these, 12 were located in non-coding regions of the genome, 122 were synonymous, 30 were nonsynonymous, and four were synonymous in one coding region and nonsynonymous in another. Dominant variants present in all sample types had an overall mean allele frequency of 99.74% (min = 98.87%, max = 99.90%). Nonsynonymous mutations and the associated amino acid changes are summarized in Table 4.

Sequencing with depth ≥ 2700 reads/position revealed evidence of iSNV in the eight clinical isolates. The detected frequencies for the minor alleles were under 5%. A total of three sites were identified as heterozygous in three coding regions along the genome: E1A, DNA polymerase, and pVII (Table 5). In the E1A coding region, a nonsynonymous iSNV was identified at nucleotide (nt) position 632 and predicted to result in amino acid change Glu25Lys in four of the eight isolates with frequencies above 3%. In the isolates obtained from liver, lung, and stool specimens, this iSNV was detected at frequencies below the set threshold. One nonsynonymous iSNV was found in the DNA polymerase coding region at nt position 6689 in the respiratory isolate. It was predicted to result in the amino acid change Pro706Ser. One additional nonsynonymous iSNV was found at nt position 16,347 in the pVII coding region in the isolate recovered from the NPS. It was predicted to result in the amino acid change Pro151Ser.

**Table 5:** Location and frequency of nonsynonymous intra-host single nucleotide variation across clinical specimen sources.

Sample Type	E1A: 632 (Glu25Lys)		Pol: 6689 (Pro706Ser)		pVII: 16,347 (Pro151Ser)	
	Ref: G	Alt: A	Ref: G	Alt: A	Ref: C	Alt: T
Kidney	95.68%	4.32%	>99.99%	-	>99.99%	-
Liver	97.03%	2.97% ^‡^	>99.99%	-	>99.99%	-
Lung	97.78%	2.22% ^‡^	>99.99%	-	>99.99%	-
NPS	>99.99%	-	96.91%	3.09%	95.47%	4.52%
Stool	98.66%	1.34% ^‡^	>99.99%	-	>99.99%	-
Urine	95.98%	4.02%	>99.99%	-	>99.99%	-
WB 1	95.91%	4.09%	>99.99%	-	>99.99%	-
WB 3	96.12%	3.88%	>99.99%	-	>99.99%	-

WB 1, WB 3: Blood specimens obtained on hospitalization days 1 and 3 at CCHMC; ^‡^ Under the minor allele frequency threshold of 3%; not recorded as true iSNV.

## 4. Discussion

We present a case of HAdV-C1 infection with severe and rapidly progressing disseminated disease of fatal outcome that provides an opportunity to identify challenges in the management of HAdV-infected patients.

Late-onset glycogen storage disease type II, also known as Pompe disease, is an autosomal recessive disorder of muscle glycogen metabolism resulting from a mutation in the acid alpha-glucosidase (AAG) gene. The late-onset phenotype results from partial enzyme function and presents as slowly progressive muscle weakness [43,44]. Individuals with Pompe disease are not reported to have impaired immunity. Prior to this infection, the child did not have a significant history of hospitalizations or other episodes of infection. At the time of his adenovirus infection, his care was discussed with his genetics medical team, who did not feel that the patient’s metabolic condition contributed to the severity of his clinical presentation. Although it cannot be ruled out that Pompe disease-related underlying conditions played a part, we speculate that the fulminant course of disease likely developed as a consequence of an early respiratory infection that rapidly disseminated after administration of immunosuppressive medications. HAdV-C1, a common cause of acute pediatric febrile respiratory infections worldwide [5,45,46,47], was detected at very high viral loads in all examined clinical specimens obtained during the patient’s short hospitalization at the referral hospital. Isolates were readily recovered from diverse clinical specimens and processed for genetic characterization at passage 2 in cell culture. Our NGS approach allowed for the identification of a strain of HAdV-C1 closely related to previously characterized strains of this genotype circulating worldwide (Figure 1). Importantly, the high sequencing depth enabled the detection with a high level of confidence of iSNVs in the viral isolates recovered from blood, liver tissue, the respiratory tract (NPS and lung tissue), urinary tract (urine and kidney tissue), and gastrointestinal tract (stool) at passage 2 in culture. Based on our set criteria for variant calling, evidence of iSNVs was found in only three coding regions (E1A, DNA polymerase, and pVII), which are involved in the initiation of viral replication and early transcription [48]. Interestingly, different variant combinations were found in different anatomical compartments.

While the existence as quasi-species and the occurrence of low-frequency genomic variants, or minor variants, have been extensively reported for RNA virus populations, data for DNA viruses are still scarce, as the diversity of genomic variants in DNA virus samples has historically been assumed to be low [49]. However, deep sequencing technologies have allowed the collection of evidence of viral population diversity in individual samples of various herpesviruses, providing the ability to describe not only an average consensus genome but also the presence of minor variants, and to evaluate the contribution of intra-host genetic diversity to disease development and progression and viral evolution [50,51,52,53].

To the best of our knowledge, this is only the second report of the detection of iSNVs in the context of HAdV infection. In 2022, Huo and colleagues described the detection of iSNVs in oral swab specimens obtained from HAdV-B3-infected patients during a 2019 outbreak of respiratory illness at a school in Mongolia [54]. Genomic sequencing revealed three “hot spots” of variation located in the DNA polymerase, terminal protein (pTP), and virus-associated (VA) RNA coding regions. Genomic sequence data analysis showed a significant reduction in the number of iSNVs detected in Hep-2 cell culture isolates compared to the corresponding original clinical specimens, indicating that some alleles are negatively selected during propagation in culture. Not having NGS data from the original specimens examined in our study is a significant limitation to describing the genomic composition of the HAdV-C1 populations present in the various specimens. The observations of Huo and colleagues suggest that our limited passaging in A549 cell culture could have altered the composition and frequency of minor variants in the sample. Consistent with the findings of Huo and colleagues, our data identified the DNA polymerase coding region as one of three coding regions with nonsynonymous mutations. However, this variant was detected only in the isolate from the NPS specimen (Table 5). Interestingly, iSNV sites were detected in the E1A coding region in all isolates except for that recovered from the NPS.

Compared to other prevalent types, HAdV-C1 has been rarely reported in cases of severe disease in immunocompetent patients [55]. While HLH has been documented as a complication of HAdV-B3 and B7 respiratory infection in children without identified primary immunodeficiencies [18,56,57,58], HAdV-C1 infection has not been reported as an HLH trigger. Although present at relatively low frequency, the impact of the detected iSNVs on viral replicative fitness and pathogenesis, as well as their functional consequences, warrants further investigation.

The contribution of immunosuppressive treatment to the severe course of disease in the patient must be taken into consideration. Since HLH is a syndrome of pathologic immune activation, the current mainstays of treatment are immunosuppressive medications [59]. It is reasonable to speculate that the administration of immunosuppressive drugs, such as methylprednisolone, Anakinra, dexamethasone, and IVIG, to modulate the exacerbated inflammatory response [60] may have dampened innate and adaptive cellular immune responses to HAdV infection, thereby increasing the risk of dissemination and contributing to disease progression. Importantly, a recent study by Galvan-Salazar and colleagues using a non-replicating HAdV-C5-derived βGal reporter vector demonstrated that dexamethasone and other anti-inflammatory drugs, such as paracetamol and ibuprofen, can increase HAdV entry into cells in vitro and in vivo in mice [61]. Whether this effect occurs in natural human infection and whether it selectively impacts infection by certain HAdV types or by certain anatomical compartments more than others warrants further investigation, as it may pose serious obstacles to the clinical management of exacerbated immune responses to HAdV infection, including HLH.

As illustrated by this case, treatment of HLH in the setting of documented infection is challenging. Immunosuppressive therapy is recommended for the treatment of HLH, but this may exacerbate the underlying infection. Therefore, we suggest that patients with infection-related HLH should first undergo treatment of the underlying infection instead of immunosuppressive therapy. If patients show clinical improvement, immunosuppression may not be necessary. If patients do not improve promptly, then immunosuppressive therapy should be given [62].

There are limited treatment modalities for disseminated HAdV disease. Cidofovir, dosed at 5 mg/kg once weekly or 1 mg/kg three times weekly, is frequently used for treatment, although strong evidence for its use is lacking. IVIG is also given at the discretion of treating physicians. For immunocompromised patients, lowering immune suppression or avoiding additional immune suppression is a critical component of the overall treatment strategy.

For reasons impossible to establish within the scope of our studies, the two administrations of cidofovir and the infusion of adenovirus VSTs the patient received during his short three-day hospitalization at CCHMC did not help control the infection. It is possible that the late administration of cidofovir could not counteract the extremely high viral load, especially given that significant organ damage had likely already occurred.

## 5. Conclusions

Two major conclusions can be drawn from the key findings of our studies: HAdV-C1 can cause lethal disseminated infections and trigger HLH in children with underlying diseases; when severe HAdV infection is combined with HLH, caution should be exercised with the use of immunosuppressive therapy.

Deep NGS of isolates obtained from various clinical specimens confirmed the presence of iSNV in regions encoding proteins implicated in viral replication. In vitro experimental work beyond the scope of our efforts for this study will be required to evaluate whether the identified mutations affect virus replication.

It is anticipated that the growing affordability and use of NGS technologies offering high depth of reads, as well as the availability of long-read sequencing platforms and oligonucleotide bait-based enrichment methods [63,64,65], will result in the rapid acquisition of more data describing the genomic composition of HAdV populations in clinical specimens in the future.

## Figures and Tables

**Table 1:** Human adenovirus C1 strains included in the phylogenetic analysis.

Strain Designation	GenBank Accession Number	Genotype	Geographical Site/ Year of Isolation
Adenoid 71	AC_000017 *	C1 (P1H1F1)	Maryland, USA/1953
9M5	OQ518270	C1 (P1H1F1)	USA/1968
A15812	JX173078	C1 (P1H1F1)	Argentina/2000
VT384	JX173082	C1 (P1H1F1)	USA/2003
VT13862	JX173086	C1 (P1H1F1)	USA/2004
2C6	OQ518311	C1 (P1H1F1)	USA/2009
3P2	OQ518280	C1 (P1H1F1)	USA/2009
5T1	OQ518269	C1 (P1H1F1)	USA/2010
9P7	OQ518349	C1 (P1H1F1)	USA/2012
C1ONP01Pr1Feb2015	OM112292	C1 (P1H1F1)	Ontario, Canada/2015
C1ONP03Cu1Jun2015	OM112288	C1 (P1H1F1)	Ontario, Canada/2015
SG09	MN513345	C1 (P1H1F1)	Singapore/2016
C1ONP05Pr1Jan2018	OM112290	C1 (P1H1F1)	Ontario, Canada/2018
QH-1665/2018	MN737436	C1 (P1H1F1)	Qinghai, China/2018
Kobe180114	LC791114	C1 (P1H1F1)	Hyögo, Japan/2018
Kobe180173	LC791122	C1 (P1H1F1)	Hyögo, Japan/2018
Kobe180476	LC791132	C1 (P1H1F1)	Hyögo, Japan/2018
8.135Hl	ON152653	C1 (P1H1F1)	Novosibirsk, Russia/2021
Kobe220083	LC791188	C1 (P1H1F1)	Hyögo, Japan/2022
AST-RII-MH147435V	PX146725	C1 (P1H1F1)	Astrakan, Russia/2023
BU-RII-MH166713V	PX255352	C1 (P1H1F1)	Buryatia, Russia/2023
YAN-RII-MH209706V	PX146724	C1 (P1H1F1)	Noyabrsk, Russia/2024
SPE-RII-MH209716V	PX146726	C1 (P1H1F1)	Saint Petersburg, Russia/2024
SPE-RII-MH209711V	PX146727	C1 (P1H1F1)	Saint Petersburg, Russia/2024
GD2467	MH558113	C104 (P1H1F2)	Guandong, China/2017
BJ09	MF315029	C108 (P1H2F2)	Beijing, China/2013
UNMC-p	OQ108498	C108 (P1H2F2)	Nebraska, USA/2021

* Prototype strain of serotype 1.

**Table 2:** Viral load in clinical specimens and results of initial molecular typing.

Clinical Specimen	Viral Load			Hexon Type	Fiber Type	Isolation in Culture
	Ct	gc/mL	Log_10_ gc/mL			
Kidney tissue ^‡^	17.6	2.55 × 10^8^	8.41	C1	C1	Yes
Liver tissue ^‡^	11.2	4.21 × 10^9^	9.62	C1	C1	Yes
Lung tissue ^‡^	13.1	1.98 × 10^9^	9.30	C1	C1	Yes
NPS	13.4	1.75 × 10^9^	9.24	C1	C1	Yes
Stool	15.4	7.32 × 10^8^	8.86	C1	C1	Yes
Urine	11.8	3.34 × 10^9^	9.52	C1	C1	Yes
WB 1	12.6	2.43 × 10^9^	9.39	C1	C1	Yes
WB 2	12.1	2.96 × 10^9^	9.47	C1	C1	Yes
WB 3	11.8	3.34 × 10^9^	9.52	C1	C1	Yes

NPS: Nasopharyngeal swab; Ct: Cycle threshold; gc: genome copies. WB 1, WB 2, WB 3: Whole blood specimens obtained on hospitalization days 1, 2, and 3 at CCHMC. ^‡^ Specimens obtained at autopsy.

**Table 3:** Quality filtering and read mapping statistics.

Library Sample	Raw Read Pairs	Post-QC		Mapping Rate		Unique Q60 Mapping		Mean Seq. Depth (Reads/Position)
		No. Retained	% Retained	No. Reads	% Reads	No. Reads	% Reads	
Kidney	381,413	379,190	99.4%	751,607	99.1%	596,985	78.7%	3103
Liver	414,082	412,344	99.6%	714,043	86.6%	535,063	64.9%	2952
Lung	453,112	450,191	99.4%	896,491	99.6%	692,186	76.9%	3666
NPS	338,566	336,813	99.5%	629,618	93.5%	504,732	74.9%	2754
Stool	429,634	427,568	99.5%	817,762	95.6%	617,218	72.2%	3442
Urine	538,303	535,434	99.5%	1,043,468	97.4%	730,995	68.3%	3984
WB 1	511,263	508,680	99.5%	1,013,218	99.6%	724,201	71.2%	3927
WB 3	411,323	408,804	99.4%	814,163	99.6%	617,931	75.6%	3296

WB 1, WB 3: Blood specimens obtained on hospitalization days 1 and 3 at CCHMC. The isolate from WB 2 was not processed for WGS.

**Table 4:** Nonsynonymous mutations identified in the genomic sequences of the examined clinical HAdV-C1 isolates through comparison with reference AC_000017.

CDS	Site	REF	ALT	Coding Variant		Notes
				Nucleotide *	Protein *	
E1A	632	G	A	c.73G>A	p.Glu25Lys	HET in WB1, Kidney, urine and WB3 (HOM-REF in all other)
	780	C	T	c.221C>T	p.Ala74Val	HOM-ALT in all isolates
	1061	G	A	c.502G>A	p.Asp168Asn	
E1B-19K/E1B 55K	2080	T	C	c.59T>C	p.Phe20Ser	HOM-ALT in all isolates
	2115	A	G	c.94A>G	p.Thr32Ala	
E1B-55K	2362	T	C	c.341T>C	p.Phe114Ser	HOM-ALT in all isolates
	2380	T	C	c.359T>C	p.Val120Ala	
pIX	3883	G	A	c.263G>A	p.Ser88Asn	HOM-ALT in all isolates
DNA pol	6689	G	A	c.2116C>T	p.Pro706Ser	HET in NPS (HOM-REF in all other)
	6861	T	G	c.1944A>C	p.Glu648Asp	HOM-ALT in all isolates
	7648	T	C	c.1157A>G	p.Asn386Ser	
	7772	C	T	c.1033G>A	p.Val345Ile	
	8363	A	G	c.442T>C	p.Leu148Leu	
DNA pol/pTP	8639	G	C	c.166C>G	p.Arg56Gly	HOM-ALT in all isolates
	8705	A	G	c.100T>C	p.Cys34Arg	
L2-pV	17,536	T	C	c.974T>C	p.Ile325Thr	HOM-ALT in all isolates
L4-100K	25,168	A	G	c.1051A>G	p.Thr351Ala	HOM-ALT in all isolates
E3-gp19K	29,042	G	A	c.268G>A	p.Val90Ile	HOM-ALT in all isolates
E3-CR1-beta	29,521	C	T	c.122C>T	p.Pro41Leu	HOM-ALT in all isolates
	29,563	C	A	c.164C>A	p.Ala55Asp	
	29,710	G	A	c.311G>A	p.Arg104His	
E3-RID-alpha	29,861	A	G	c.34A>G	p.Ile12Val	HOM-ALT in all isolates
L5-fiber	31,320	A	G	c.220A>G	p.Lys74Glu	HOM-ALT in all isolates
	31,696	A	G	c.596A>G	p.Asn199Ser	
	32,115	C	A	c.1015C>A	p.His339Asn	
	32,340	C	T	c.1240C>T	p.His414Tyr	
	32,425	G	A	c.1325G>A	p.Arg442Lys	
	32,514	A	G	c.1414A>G	p.Ser472Gly	
	32,681	A	T	c.1581A>T	p.Glu527Asp	
E4-ORF6/7	33,164	T	A	c.264A>T	p.Arg88Ser	HOM-ALT in all isolates
	33,178	T	C	c.250A>G	p.Asn84Asp	
	33,226	C	T	c.202G>A	p.Glu68Lys	
E4-ORF4	34,205	C	A	c.199G>T	p.Ala67Ser	HOM-ALT in all isolates
E4 -ORF3	34,670	A	C	c.98T>G	p.Ile33Arg	HOM-ALT in all isolates

* Human Genome Variation Society (HGVS) Nomenclature: “c.” description of a variant relative to a coding DNA reference sequence; “p” predicted consequences on the protein level. Location of variants based on the NCBI HAdV-C1 reference genome (AC_000017.1). Reference (REF) and alternate (ALT) nucleotides are displayed as observed in the ‘+’ strand of the genome. Variant sites within each gene are displayed using HGVS notation (c. for coding DNA and p. for protein). Variant notes indicate the nucleotides present at the position based on reads from the same sample: heterozygous (HET) containing both the reference and alternate nucleotide, homozygous reference (HOM-REF) containing no alternate nucleotide, and homozygous alternate (HOM-ALT) containing only the alternate nucleotide (>99.9%).

## Data Availability

All sequence data generated for this body of work are available under BioProject accession number PRJNA1046048 in the NCBI BioProject database (https://www.ncbi.nlm.nih.gov/bioproject, accessed on 1 May 2026).

## Abbreviations

The following abbreviations are used in this manuscript:

HAdV	Human adenovirus
HLH	Hemophagocytic lymphohistiocytosis
iSNV	Intra-host single-nucleotide variation
NPS	Nasopharyngeal swab
WB	Whole blood
ITR	Inverted terminal repeat
